# Arterial Glyceryl Trinitrate in Acute Ischemic Stroke after Thrombectomy for Neuroprotection (AGAIN): Rationale, design and protocol for a prospective randomized controlled trial

**DOI:** 10.1186/s12877-022-03506-5

**Published:** 2022-10-17

**Authors:** Jie Gao, Zhe Cheng, Shangqian Jiang, Melissa Wills, Alexandra Wehbe, Gary B. Rajah, Xiaokun Geng, Yuchuan Ding

**Affiliations:** 1 Department of Neurology and Stroke Center, Beijing Luhe Hospital, Capital Medical University, No. 82 Xinhua South Road, Tongzhou District, Beijing, 101149 China; 2 Luhe Institute of Neuroscience, Capital Medical University, Beijing, 101100 China; 3 Department of Neurosurgery, Wayne State University School of Medicine, 550 E Canfield, Detroit, MI 48201 USA; 4 Harvard T.H. Chan School of Public Health, Boston, MA 02138 USA; 5 Department of Neurosurgery, Munson Healthcare, Traverse City, MI USA; 6 Department of Neurosurgery, Jacobs School of Medicine and Biomedical Sciences, Department of Neurosurgery, University at Buffalo, Gates Vascular Institute at Kaleida Health, Buffalo, NY USA

**Keywords:** Nitric oxide, Neuroprotection, Large vessel occlusion, Randomized controlled trial

## Abstract

**Background:**

Although endovascular recanalization therapy demonstrates robust clinical efficacy in acute ischemic stroke (AIS), not all victims of these cerebrovascular accidents can benefit from it and achieve a favorable prognosis after successful reperfusion. Therefore, alternative neuroprotective strategies are urgently needed for AIS patients after vessel recanalization. Nitric oxide (NO) levels are low after AIS and NO donor drugs may be neuroprotective against cerebral ischemia–reperfusion injury. Glyceryl trinitrate (GTN), often used in the clinic as a NO donor, may provide a novel neuroprotective strategy. This rationale, design, and protocol for a prospective pilot study plans to explore the preliminary safety, feasibility, and neuroprotective benefits of Arterial Glyceryl Trinitrate in Acute Ischemic Stroke after Thrombectomy for Neuroprotection (AGAIN).

**Methods:**

AGAIN, a prospective RCT, is proposed for AIS patients after mechanical thrombectomy. Subjects will be randomly assigned in a 1:1 fashion (*n* = 40) to either the control group or the intervention group. Participants assigned to the intervention group will be administered 800 μg GTN in the catheter immediately after recanalization, whereas those in the control group will be administered the same volume of normal saline. All participants from either group will be given concurrent treatment with standard of care therapies in accordance with the current guidelines for stroke management. The primary outcome is safety [symptomatic intracranial hemorrhage (ICH), hypotension, neurological deterioration, ICH, fatal ICH, as well as headache, tachycardia, emesis, and seizures], whereas secondary outcomes included changes in poststroke functional outcomes, infarction volumes, and blood nitrate index detection.

**Discussions:**

This study is a prospective randomized controlled trial to test the safety and efficacy of intra-arterial GTN in AIS patients after endovascular therapy. The results from this study will give insight for future GTN studies and new neuroprotective strategies for future AIS treatment strategies.

**Trial registration number:**

ChiCTR2100045254. Registered on March 21, 2021.

## Background

Acute ischemic stroke (AIS) is a severe disease and devastating public health concern– it carries a substantial socioeconomic burden with remarkably high morbidity and mortality rates [1, 2]. New medical advances, including the new generation of stent-retrievers, have been successful in improving the rates of revascularization after AIS and in expanding the temporal intervention window for patients who suffer large vessel occlusions (LVO). Advances such as these have lowered rates of disability and mortality and improved post-stroke outcomes. However, despite this cutting-edge technology, there is an apparent discrepancy between the use of effective recanalization and the ability to regain pre-stroke functional abilities. Indeed, not only does the mortality rate remain at 15.3%, but only 46% of individuals with anterior circulation AIS who are treated with endovascular therapy achieve functional independence after 90 days [3]. Furthermore, there is significant heterogeneity across studies reporting the long-term outcomes of thrombectomy and several aspects of this technique are yet to be optimized, such as the time window for effective treatment [3–6]. As a result of these uncertainties, attention must be given not only to advances in vascular recanalization, but also to neuroprotection in the post-stroke setting in order to maximize the potential of these emerging therapies. Exploring fast and effective neuroprotection strategies to supplement mechanical thrombectomy will become a key strategy for improving the prognosis of the post-stroke patient [7]. We hypothesized that glyceryl trinitrate (GTN), a donor of nitric oxide (NO), may benefit patients in the context of AIS after endovascular recanalization.

The efficacy of reperfusion after ischemic stroke is limited by post-ischemic inflammatory changes, including reactive oxygen species (ROS) formation and accumulation, which may cause apoptosis as well as unregulated and pathologic cell death [8]. ROS and nitrogen species are highly destructive products of ischemic-reperfusion injury and that can exacerbate neuronal death through DNA damage, lipid peroxidation, and protein oxidation [9]. NO plays a pivotal part in hypoxic signaling and, at physiologic and therapeutic levels, exerts powerful cytoprotective effects after ischemia and reperfusion in a variety of tissues, including neurologic tissue [10, 11]. Though the mechanism remains elusive, various studies have reliably exhibited reduced cytoprotection when subjects were administered NO scavengers or in experiments where NO synthase was knocked out or inhibited [12–14]. ROS formation induced by reperfusion contributes to a decrease in the bioavailability of NO, thus limiting its ability to promote various protective mechanisms after stroke, such as vasodilation, inflammation inhibition, and preservation of endothelial integrity under hypoxic conditions [15]. NO donors are a category of drugs, independently of its endogenous sources, whose common mechanism is a propensity to release NO or a NO-related substance in vitro or in vivo [16]. Abundant preclinical research suggests NO donors can safely decrease infarction volume, increase cerebral blood flow, and better functional outcomes in AIS in both transient and permanent stroke models [17]. Additionally, NO donors’ neuroprotective effect has been shown to alleviate ischemia–reperfusion injury at various levels in multiple different ways, including modifying cellular oxidative status, inhibiting activity of monocytes, and reducing primary hemostasis [18].

GTN is a vasodilator approved by the Food and Drug Administration which acts as an NO donor and has been experimented with for neuroprotection in AIS. Transdermal GTN has been shown to lower blood pressure without any deleterious effects on platelet function or changes to the middle cerebral artery (MCA) blood flow velocity or to regional cerebral blood flow in patients suffering an AIS [19–22]. A recent RCT with a larger sample size studied the efficacy of transdermal GTN in high blood pressure management in AIS (ENOS) similarly showed the GTN’s ability to lower blood pressure, but did not indicate an improvement in functional outcome following AIS [23]. However, NO donors’ neuroprotective effects following ischemic–reperfusion injury have consistently shown to be dependent on the dose, location, source and environment [24]. Considering these parameters and GTN’s short half-life, administering GTN as an arm or chest patch might not reach the cerebrovascular system in a way that enables it to confer significant or reliable benefits. Recently, arterial administration of GTN in MCAO animal models demonstrated a neuroprotective effect in AIS [24]. Rather, intra-catheter GTN administration might be a quicker, more effective method to allocate the full potential of GTN. To our knowledge, there is a paucity of literature regarding GTN’S efficacy and safety as an adjunctive method of neuroprotection to endovascular recanalization in AIS. Thus, this single-center, prospective randomized controlled study serves to assess the safety, feasibility, and preliminary efficacy of intra-catheter GTN administration post-mechanical thrombectomy.

## Methods/design

This study was registered on www.chictr.org.cn in April 2021 (ID: ChiCTR2100045254) and approved by the ethics committee of Luhe Hospital, Capital Medical University, Beijing, China.

### Study design

AGAIN is a prospective, randomized, controlled, single-center study designed to evaluate the safety and efficacy of GTN administration after mechanical thrombectomy in patients suffering an AIS within 6 h of symptom onset.

### Participants and screening

**Fig. 1:**
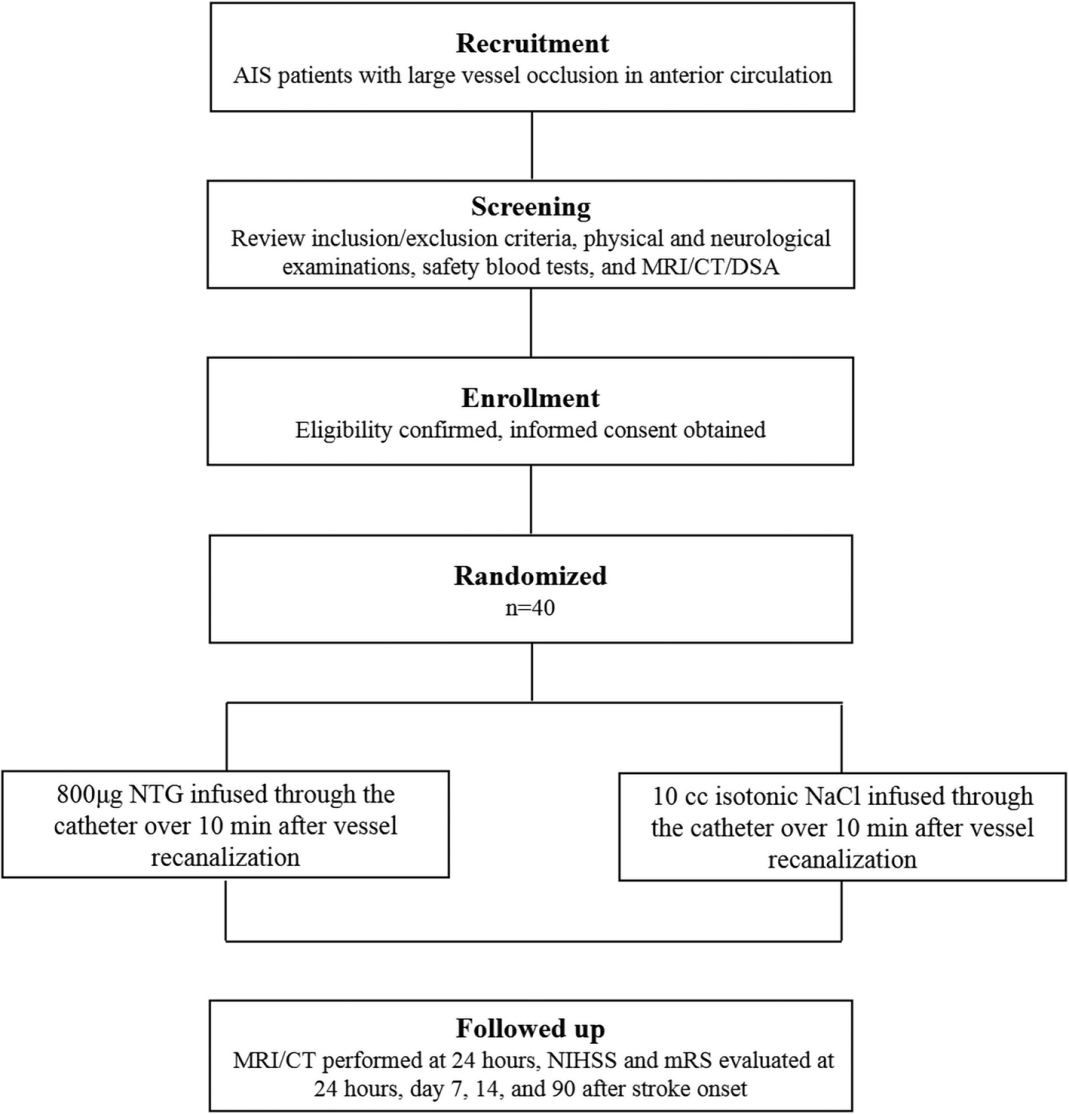
Flowchart of the study

Eligible patients will be enrolled consecutively after undergoing endovascular therapy for AIS in the Beijing Luhe Hospital Stroke Center, Capital Medical University. Participants for this study will be recruited from the stroke center (the Stroke Intervention & Translational Center, SITC) in Beijing Luhe Hospital. Informed consent, which includes an explanation of potential benefits and risks, will be obtained from each participant. Once all participants have read the participant information sheet and provided written informed participation consent, they will be evaluated against eligibility criteria and, if eligible, will be enrolled in our study. Consent will be obtained by an appointed legal representative should the participant lack decision-making capacity, see Fig. 1.

*The inclusion criteria include*: 1) AIS patients with anterior circulation LVO who received endovascular therapy, 2) age between 18 and 80 years, 3) within 6 h from symptom onset to puncture, 5) baseline ASPECT score 6–10, 6) baseline NIHSS score 6–25.

*Exclusion criteria are as follows*: 1) lack of vessel recanalization (TICI score = 0), 2) evidence suggesting spontaneous vessel recanalization, 3) rapid spontaneous amelioration of neurological function to an NIHSS < 6 after vessel recanalization, 4) systolic blood pressure < 120 mmHg before GTN administration, 5) blood glucose < 2.7 or > 22.2 mmol/L, 6) evidence of coagulation disorders in laboratory studies, 7) pregnancy, 8) pre-stroke modified Rankin Scale (mRS) > 2, 9) no informed consent was obtained, 10) enrollment in other clinical studies within 3 months.

A physician unaffiliated with the study will monitor the safety and health status of participants. A safety quality control conference was held by the GCP committee twice a year and on an as needed basis. The safety of subjects was continuously monitored by Luhe Hospital Ethics Committee.

### Randomization and blindness

The participants will provide consent and assigned randomly in a 1:1 (*n* = 40) fashion to the two treatment groups with the use of computer-generated randomization. Interventionalists, outcome assessors, and investigators involved in data collection and analysis for the trial will be blinded to this assignment with the use of opaque envelopes.

### Interventions

Stroke patients that satisfy inclusion criteria will be assigned randomly to either the control group or experimental group. All patients will also receive standard stroke treatment, including antiplatelet or anticoagulation medications, antihypertensive medications, hyperlipidemia and hyperglycemia control, and neurotrophic treatment. In experimental group, 800 μg NTG will be diluted using 10 cc isotonic NaCl solution administered via catheter over 10 min after vessel recanalization. The control group will receive 10 cc isotonic NaCl administered via the catheter over 10 min.

### Outcomes and assessment procedures

Participant demographic information, stroke risk factors, occlusion site, and procedure of mechanical thrombectomy will be recorded. On hospital admission, ASPECTS (Alberta Stroke Program Early CT Score) and NIHSS (National Institute of Health stroke scale) scores will be determined and recorded for further analysis. Magnetic resonance imaging (MRI) or computerized tomography (CT) will be completed 24 h after mechanical thrombectomy. NIHSS and mRS scores will also be determined at baseline and at 24 h, day 7, 14, and 90 after stroke onset.

*Primary Outcomes (Safety Assessment)*. The primary safety consideration of AGAIN is the risk of symptomatic intracranial hemorrhage (ICH). The secondary safety outcomes include: 1) episodes of hypotension requiring clinical treatment during GTN administration; 2) neurological deterioration is defined as NIHSS score increasing more than 4 point from baseline to day 7; 3) ICH, fatal ICH; 4) death regardless of cause during the study period; 5) headache, tachycardia, emesis, and seizures.

*Secondary Outcomes (Efficacy Assessment)*. The main parameter to assess efficacy of AGAIN is functional independence on day 90 post-intervention (as determined by scores between 0 to 2 on the mRS scale, which are representative of functional independence), infarct volumes, NIHSS scores, and blood nitrate index detection [cyclic guanosine monophosphate (cGMP), L-arginine, and L-citrulline)]. The infarct volume is measured using the Siemens Syngo with Diffusion-Weighted Imaging (DWI) by the image tool (ROI) (this is done by outlining the infarcted area on each section in order to calculate the area and subsequently by multiplying this value from each layer by its respective thickness).

### Estimation of sample size

Intra-catheter administration of GTN in AIS patients after mechanical thrombectomy is our first attempt to address the effect of GTN in AIS patients with endovascular therapy. There is no data available for reference. AGAIN is a pilot study to examine the study design, methods, procedures, inclusion criteria, and operational strategies. The results will be used to estimate the sample size and conduct a power analysis for a phase-2 trial. Based on two other protocols published in Frontiers in Neurology [25, 26], 10 to 20 participants per group may be adequate to assess the initial safety and feasibility trends of intra-catheter administration of GTN. Therefore, a similar number of sample of total 40 patients was selected in this pilot study.

### Statistical analyses

The endpoint event in the present study will be assessed using the concept of intention-to-treat (ITT). In the event that no differences in incidence of adverse events or functional outcomes are found at 90 days when comparing the experimental and control groups, our team will proceed onto a phase-2 trial.

The Kolmogorov–Smirnov test for normality and the equal variance will be conducted prior to any other statistical analysis methods. For continuous variables with normal distribution, a 2-sided *t* test for independent samples will be applied in order to detect differences between the groups. A Mann–Whitney U test will be performed for continuous variables that have a non-normal distribution. For categorical variables, χ2 or Fisher exact tests will be used when fitting. A *p* value of < 0.05 will be the threshold to determine statistical significance. Statistical analysis will be performed on SPSS 22.0.

## Discussion

As the population continues to age, the incidence of cerebrovascular events and a concurrent need to develop neuroprotective therapies will increase significantly [1]. At present, mechanical thrombectomy is the most effective method of revascularization for patients who suffer an AIS with LVO. However, clinical use of this therapy is restricted by several parameters, including a narrow temporal window for its use, potential complications, as well as reperfusion injuries such as brain edema and hemorrhagic transformation [27, 28]. In order economize time for mechanical recanalization, reduce reperfusion injury, and maximize the potential of existing stroke therapies, investigating fast and effective neuroprotective strategies has become an essential goal in improving the prognosis post AIS.

Recently, stroke investigators have examined several novel methods for their potential neuroprotective properties in AIS. However, most neuroprotectants have only shown reliable benefits in stroke models using animals, and have been unsuccessful in clinical translation to human subjects [7, 29, 30]. Combining such neuroprotective techniques with intravenous or endovascular therapy is considered to be an important impending phase in the development of effective treatment strategies in AIS [31]. The recent developments in mechanical thrombectomy to treat AIS have resulted in high rates of successful vessel recanalization and have paved the way for promising new opportunities to effectively apply supplementary neuroprotective therapies to patients after reperfusion and to expand the time frame for effective reperfusion [29]. Studies have sought to investigate various mechanisms of neuroprotection as potential adjuncts to mechanical thrombectomy, such as the inhibition of excitotoxic cell death in acute ischemia. Recently, for example, the ESCAPE-NA1 clinical trial found that nerinetide, a neuroprotectant that disrupts excitotoxic post-synaptic density protein 95 (PSD-95), resulted in better functional prognosis and decreased mortality among AIS patients who received mechanical thrombectomy [32]. Additionally, also when merged with mechanical thrombectomy, short-duration intraarterial selective cooling infusion selectively administered into an ischemic region appears to be safe and is associated with reduced infarct volume patients suffering an AIS [33]. Furthermore, oxygen supplementation, a traditional neuroprotectant, demonstrates the ability to reduce reperfusive and inflammatory injury into the penumbra, reduce cell damage, and stimulate neural recovery and neuroplasticity post AIS. [34]. Accordingly, our previous clinical study showed that high-flow normobaric oxygen (NBO) therapy post-mechanical thrombectomy is effective in reducing infarct volumes, improving functional prognosis, and decreasing mortality in patients suffering an AIS affecting anterior circulation [35]. These works highlight the many potential avenues of neuroprotection and further suggest a promising combination of reperfusion and neuroprotective approaches for the care of AIS patients. In addition to neuroprotectant, adjunct intra-arterial alteplase after successful recanalization might be also effective for tissue salvage [36]. Application of various drugs after vessel recanalization is promising for stroke treatment to improve functional prognosis after mechanical thrombectomy in the future.

The neuroprotective effect of NO donors has been found to prevent ischemia–reperfusion (I/R) injury after AIS in preclinical studies [18]. GTN is one of the most extensively used exogenous NO donors clinically, but its application after endovascular recanalization has not been reported yet. Transdermal and sublingual GTN has failed to confer improvements in functional outcome in AIS patients [19–23]. The neuroprotective effect of NO donors are suggested to be consistently dependent on the dose, location, source and environment [24]. Due to cascade expansion of inflammatory responses during the whole process of I/R injury, early intra-arterial administration of GTN after vessel recanalization (during the early stage of I/R injury) may be the best method to prevent ischemic-reperfusion injury and exert its clinical neuroprotective benefits. Additionally, arterial administration of GTN has been demonstrated to be neuroprotective in MCAO animal model [24]. Therefore, it is possible that intra-arterial administration of GTN after vessel recanalization would most effectively enable this compound to reach target sites and have the greatest therapeutic effect. We will determine the safety of intra-arterial GTN and its prospective ability to improve functional outcomes in patients who suffer an AIS. This sequence may best enable us to assess the benefits and clinical transformation of GTN and mechanical thrombectomy in AIS patients.

There are certain limitations of the proposed study. First, the trial will be conducted at single center and has a small sample size, which may influence the generalizability of the conclusions that may be drawn and potentially invites illegitimate results to the study. Second, the target dosage of GTN still needs optimization in the context of its use after mechanical thrombectomy. The dose of GTN was decided according to the coronary artery research in which 200-500ug GTN was given through a catheter [37–39] and clinical experience of GTN used in cardiovascular and cerebrovascular diseases intervention therapy.

AGAIN proposes to ascertain the safety, feasibility, and, secondarily, the efficacy of intra-arterial GTN combined with mechanical thrombectomy in patients who suffer an AIS. The preliminary data will provide the parameters for clinical trials in the future.

## Data Availability

Datasets created during and/or analyzed for this study will be confidential. However, after publication of peer-reviewed journal articles, congregated data will be obtainable from the corresponding author upon request.

## Abbreviations

AIS: Acute ischemic stroke
LVO: Large vessel occlusion
IRI: Ischemia–reperfusion injury
GTN: Glyceryl trinitrate
NIHSS: National Institutes of Health Stroke Scale
MRS: Modified Rankin scale
sICH: Symptomatic intracranial hemorrhage
BI: Barthel index
CT: Computed tomographic
MRI: Magnetic resonance imaging
Mtici: Modified Thrombolysis in Cerebral Infarction
